# The American Year-Book of Medicine and Surgery for 1903

**Published:** 1903-10

**Authors:** 


THE AMERICAN YEAR-BOOK OF MEDICINE AND SURGERY FOR 1903. —A yearly digest of scientific progress and authorative opinions in all branches of medicine and surgery, drawn from journals, monographs, and text-books of the leading American and foreign authors and investigators. Arranged, with critical editorial comments by eminent American specialists, under the editorial charge of George M. Gould, A. M., M. D., in two volumes. Volume I, including General Medicine. Octavo, 700 pages, fully illustrated. Volume II, General Surgery. Octavo, 670 pages, fully illustrated. Philadelphia, New York, London: W. B. Saunders & Co. 1903. Per volume: Cloth, $3.00 net; half Morocco, $3.75 net.

We know of no similar publication, either American or foreign, that can compete in any way with this excellent year-book, published by W. B. Saunders & Co. It is not an indiscriminate collection of extracts and clippings but a digest of scientific progress in all branches of medicine and surgery, embracing every new theory and scientific discovery worthy of consideration. Dr. Gould was wise in his selection of eminent authorities as assistants, the names of which alone are sufficient guarantee of its value. Volume I contains General Medicine, Volume II General Surgery, and can be obtained separately if desired. The illustrations are excellent, there being eleven full-page inserts, besides many text cuts. We strongly recommend Saunders' American Year-Book. R. & L.

